# Supplementary material for: Heavy Metal Effects on Biodiversity and Stress Responses of Plants Inhabiting Contaminated Soil in Khulais, Saudi Arabia
Source: Biology (Basel). 2022 Jan 20;11(2):164. doi: 10.3390/biology11020164 (PMC8869145; doi:10.3390/biology11020164)
Supplement: Supplementary file 1 [file biology-11-00164-s001.zip › biology-1551642-supplementary.pdf]

Table S1

List of the recorded species in the different sites with their, families, relative densities (RD) and frequencies (F) in the different sites.

families	Species	Site 1		Site 2		Site 3		Site 4		Site 5	
		**RD**	**F**	**RD**	**F**	**RD**	**F**	**RD**	**F**	**RD**	**F**
Aizoaceae	*Blepharis attenuate* Napper	0	0	0	0	0	0	0	0	3.4	10
	Aizoon canariense L.	0	0	0	0	0	0	0	0	1.3	8
Aizoaceae	*Trianthema portulacastrum* L.	1	0	8.3	25	0	0	2	13	0.9	3
Amaranthaceae	*Aerva javanica* (Burm. f.) Juss. ex Schul	0	0	0	0	0	0	1.6	22	3.4	6
	Amaranthus graecizan L.	6.7	60	6.1	55	3.4	21	1.1	7	0	0
	Amaranthus viridis L.	5.4	65	5.7	45	3.2	19	1.4	5	0	0
	***Amaranthus retroflexus* L.**	16.7	100	15.3	95	6.7	36	2.6	25	0	0
Asclepiedaceae	*Calotropis procera* (Aiton) W.T.Aiton	1.9	15	1.2	10	1.1	10	2.1	23	3.1	12
	Leptadenia pyrotechnica (Forssk.) Decne.	0	0	0	0	0	0	1.8	0	2.1	7
Boraginaceae	*Heliotropium bacciferum* Forssk.	0	0	1	5	1.2	13	1	11	1.9	5
Capparaceae	*Mareua oblongifolia* (Frossk.) A.Rich.	0	0	0	0	0	0	1.1	10	1.5	6
	Diptergium glaucum Decne.	0	0	0	0	1.3	11	2.4	11	3.1	12
	Cadaba farinosa Frossk.	0	0	0	0	0	0	1.3	0	1.2	8
	Cadaba glandulosa Frossk.	0	0	0	0	0	0	1.1	0	1.2	3
Chenopodiacea	*Salsola tetrandra* Forssk	0	0	1	7	1.1	9	1	9	0.8	7
	Suaeda monica Frossk.ex J.F. Gmel.	1.5	20	1.3	13	2.3	10	1.1	10	1.2	10
Convolvulaceae	*Convolvulus deserti* Hochst. & Steud.	0	0	0	0	0	0	0	0	0.7	6
	Ipomoea pes-caprae	2.4	3	0	0	0	0	0	0	0	0
Cucurbitaceae	*Citrullus colocynthis* (L.) Schrad.	0	0	0	0	0	0	0	0	0.9	5
Cyperaceae	*Cyperus conglomerates* Rottb.	0	0	0	0	1.4	11	0	0	0.4	2
Fabaceae	*Acacia hamulosa* Benth.	0	0	0	0	0	0	0	0	3.6	7
	Acacia tortilis (Forssk.) Hayne	0	0	0	0	0	0	2.3	7	3.7	7
	Astragalus vogelii (Webb) Bornm.	0	0	0	0	0	0	0	0	0.5	3
	Indigofera spinosa Frossk	0	0	0	0	0	0	0	0	1.7	14
	Prosopis Juliflora (Sw.) DC.	18.2	90	16.8	85	6.3	22	1.1	2	2.3	11
	Senna italica Mill.	0	0	0	0	1.1	9	2.8	16	3.6	15
Malvaceae	*Abutilon pannosum* (G. Forst.) Schltdl.	0	0	0	0	0	0	0	0	1.1	3
Nyctaginaceae	*Boerhavia diffusa* L.	0	0	0	0	0	0	0	0	1.2	8
Poaceae	*Cynodon dactylon* (L.) Pers.	6.5	11	0	0	0	0	0	0	0	0
	Echinochloa colona (L.) Link	8.9	50	7.4	45	6.8	24	2.1	12	0	0
	Leptochloa fusca (L.) Kunth	9.1	60	6.8	47	5.7	19	1	4	0	0
	Panicum turgidum Forssk.	0	0	0	0	1.6	2	2.4	15	1.4	11
	Pennisetum divisum (J.F. Gmel.) Henrard	2.7	20	2.7	17	2.4	24	1.7	6	1.1	10
	Phragmites australis (Cav.)Trin ex Steud	6.7	25	6.4	23	2.3	14	0	0	0	0
	Stipa capensis Thunb.	0	0	0	0	0	0	1.3	12	1	13
Polygalaceae	*Polygala erioptera* DC.	0	0	0	0	0	0	0	0	1	2

Portulacaeae	*Portulaca oleraceae* L.	6.2	45	5.9	43	0	0	1	4	0	0
Resedaceae	*Ochradenus baccatus* Del.	0	0	0	0	0	0	0	0	3.1	13
Solanaceae	*Solanum incanum* L.	0	0	0	0	0	0	0	0	3.4	16
Tamaricaceae	*Tamarix nilotica* (Ehrenb.) Bunge	2.3	20	2.1	21	2.1	13	1	3	1.1	12
	Fagonia mollis Delile.	0	0	0	0	0	0	0	0	2.7	5
Zygophyllaceae	*Tribulus macropterus* Boiss.	0	0	1.1	8	0	0	1.1	17	1.3	14
